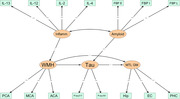# Interplay of inflammatory, vascular, and Alzheimer’s disease biomarkers in cognitively unimpaired older adults

**DOI:** 10.1002/alz.091623

**Published:** 2025-01-09

**Authors:** Batool Rizvi, Jenna N. Adams, Alison Bamford, Mithra Sathishkumar, Soyun Kim, Liv McMillan, Adam M. Brickman, Mark Mapstone, Elizabeth A. Thomas, Michael A. Yassa

**Affiliations:** ^1^ University of California, Irvine, Irvine, CA USA; ^2^ Columbia University, New York, NY USA

## Abstract

**Background:**

Neuroinflammatory processes, assessed by cytokines such as interleukins, are implicated in vascular disease and amyloid‐β (Aβ) burden. White matter hyperintensities (WMH), markers of small vessel cerebrovascular disease, are associated with memory impairment and Alzheimer’s disease (AD)‐related cortical atrophy. Here, we used structural equation modeling (SEM) to test whether inflammatory markers are related to markers of AD pathology and neurodegeneration through their impact on WMH.

**Method:**

A sample of 118 older adults without dementia (mean age (SD) = 70.8 (6.22) years, age range: 59.8‐86.4 years, 77 (65%) women) were included. Regional WMH volumes were derived from T2‐FLAIR images using ANTs, parcellated by a vascular territory atlas. Aβ was assessed with 18F‐florbetapir‐PET, with SUVR was extracted from composite ROIs reflecting early, intermediate, and late regions of deposition. Medial temporal lobe (MTL) volume/thickness measures including averaged bilateral hippocampal volumes, entorhinal cortex thickness, and parahippocampal cortex thickness, were derived using FreeSurfer v.6.0. A set of inflammatory markers (IL‐2, IL‐4. IL‐6, IL‐8, IL‐10, IL‐12, IL‐13) and phosphorylated tau (P‐tau217, P‐tau181) were measured in plasma. We tested the relationships among inflammation, amyloid‐β, WMH, P‐tau, and MTL gray matter using structural equation modeling (SEM) and confirmatory factory analysis (CFA).

**Result:**

Model fit indices demonstrated acceptable model fit: χ2 (84) = 104.328, p = 0.066; comparative fit index (CFI) = 0.982; Tucker‐Lewis index (TLI) = 0.977; Root Mean Squared Error of Approximation (RMSEA) = 0.070 (90% CI: 0.021, 0.104); and Standardized Root Mean Square Residual (SRMR) = 0.107. Inflammation was associated with WMH (b=0.430, β=0.403, p=0.031), but not with amyloid‐β burden (b=0.099, β=0.099, p=0.549). Only amyloid‐β was related to phosphorylated tau (b=0.535, β=0.462, p<0.001). Both WMH and phosphorylated tau burden were associated with reduced MTL gray matter (WMH: b=‐0.228, β=‐0.231, p=0.049; P‐tau: b=‐0.233, β=‐0.251, p=0.031).

**Conclusion:**

Our study suggests that inflammation promotes AD‐related neurodegeneration through its impact on vascular disease whereas amyloid appears to operate through tau in its effect on atrophy. The study highlights the possible ways in which inflammatory and vascular pathways converge with amyloid pathways to drive AD‐related neurodegeneration.